# Modeling of a Passive-Valve Piezoelectric Micro-Pump: A Parametric Study

**DOI:** 10.3390/mi11080752

**Published:** 2020-07-31

**Authors:** Akam Aboubakri, Vahid Ebrahimpour Ahmadi, Ali Koşar

**Affiliations:** 1 Faculty of Engineering and Natural Science, Sabanci University, Istanbul 34956, Turkey; akamaboubakri@sabanciuniv.edu (A.A.); vahid@sabanciuniv.edu (V.E.A.); 2 Sabanci University Nanotechnology and Applications Center (SUNUM), Sabanci University, Tuzla, Istanbul 34956, Turkey; 3 Center of Excellence for Functional Surfaces and Interfaces for Nano-Diagnostics (EFSUN), Sabanci University, Istanbul 34956, Turkey

**Keywords:** micro-electro-mechanical systems (MEMS), piezoelectric micro-pump, passive-valve, numerical modeling

## Abstract

Piezoelectric micro-pumps offer many applications and could provide considerable flow rates in miniature systems. This study parametrically investigates the effects of major parameters, namely the length, width and attack angle of valves, piezoelectric length, and applied voltage. The results show that these parameters significantly affect the performance of the designed micro-pump. Even though increasing the piezoelectric length and operating voltage raise the flow rate, the modification of valve dimensions is more efficient since these parameters do not rely on any external power. According to the obtained results, as the length of the working valves increases, the provided flow rate becomes larger. There is an optimum condition for the width and attack angle of the valves. This optimum width is not dependent on the flow rate. With the use of the attack angle and the length of the valves as design parameters, the studied design shows promising results.

## 1. Introduction

Over the past few decades, the developments in the Micro-electro-mechanical Systems (MEMS) technology have realized the implementation of microfluidic devices to a wide range of applications such as drug delivery systems [1], micro-mixing [2] and particle manipulation [3], among others. Micro-pumps are one of the most frequently used devices, which supply the energy required to drive a fluid through microfluidic passages. Thus far, many micro-pump designs have been accomplished with various structures and operating mechanisms [4]. These designs change from one application to another based on the requirements and working conditions [5]. 

According to the operation mechanism, micro-pumps are categorized in two branches: mechanical and non-mechanical micro-pumps. In a mechanical micro-pump, the flow is driven inside the channel by using a moving mechanical part. On the other hand, there is no moving mechanical part in non-mechanical micro-pumps, which are capable of converting the non-mechanical energy into the mechanical energy. For instance, magneto hydrodynamic (MHD) micro-pumps involve the flow of electrically conducting fluid under electric and magnetic fields [6]. These micro-pumps can only pump conductive fluids. In an innovative manner, the magnetic actuation of ferrofluids was also used in micro-pumping by taking the advantage of dynamic magnetic fields [7,8]. Another example of a non-mechanical micro-pump is electro-hydrodynamic (EHD) micro-pumps, which induce fluid flow through the action of electrostatic forces on dielectric liquids [9].

In mechanical micro-pumps, the physical actuator propels the fluid from the inlet to the outlet. For example, electrostatic and electroactive polymer composite micro-pumps depend on the Columbic attraction of two oppositely charged bodies to induce displacement or exert a force [10]. Another example of mechanical micro-pump is a thermal actuation micro-pump. These pumps are typically based on thermo-pneumatic, shape memory alloy, or thermally expandable polymer mechanisms [11].

Another well-known mechanical micro-pump is a piezoelectric micro-pump. Generally, piezoelectric micro-pumps are divided into two categories: with and without valves. Valve-less piezoelectric micropumps were studied experimentally and numerically [12,13,14]. The piezoelectric micro-pumps equipped with valves consist of a pumping chamber with a diaphragm (pumping membrane), a microchannel and two check valves [15,16,17]. The main advantages of piezoelectric micro-pumps include low noise [18] and energy consumption [19]. Furthermore, the excited piezoelectric actuators have a large actuation force [20]. The implemented voltage or frequency to the piezoelectric device adjusts the flow rate in piezoelectric micro-pumps. Generally, piezoelectric actuators do not require any high voltage to operate. In addition, small size, rapid response, and simple structure are other features of this type of micro-pumps [21]. However, their fabrication process is rather complex [20].

The first piezoelectric pump was fabricated using micromachining methods by Van Lintel et al. [22]. The fabricated pump consisted of a pumping chamber, silicon check valves and a thin glass membrane actuating along with a piezoelectric wafer. This pump was able to provide flow rate and back pressure of 8 μL/min and 9.8 kPa, respectively, when 125 V voltage and 1 Hz frequency were applied. Thereafter, Esashi et al. [23] developed a pump which was able to provide 15 μL/min and 6.4 kPa maximum flow rate and back pressure at 90 V voltage and 30 Hz frequency, respectively. According to the study by Olosson et al. [24], the micro-pump efficiency could be increased by embedding two chambers. The micro-pump fabricated by Schabmueller et al. [25] with passive valves provided 1500 μL/min flow rate and 1 kPa back pressure. Yu-feng et al. [26] studied a parallel dynamic micro-pump comprising a valve, electro-magnetic coil and diaphragm. They applied a frequency of 100 Hz with a current of 0.3 A and reported a 30-μm displacement in diaphragm and 6-μL/s maximum flow rate. Shen et al. [27] integrated electromagnetic actuation (2W) and reciprocating PMMA ball valve to characterize the fabricated micro-pump. The obtained backpressure and flow rate were 35 kPa and 6 mL/min, respectively, for 20 Hz resonant frequency.

Even though there are numerous experimental studies on piezoelectric micro-pumps in the literature, the number of numerical and modeling studies is rather limited. Unlike the typical designs, where the valves are fully closed and perpendicular to the fluid flow direction, the valves are not fully closed in this design, where there is an attack angle between the valves and fluid flow. This study includes the experimental validation of numerical simulations using the measured flow rates of a micro-pump from the literature and is the first study, which investigates the effect of various parameters on the performance of the introduced piezoelectric micro-pump. The considered parameters are the length, width and attack angle of the valves, piezoelectric length, and applied voltage on the piezoelectric device.

## 2. Methodology

### 2.1. Working Theory

Actuators are one of the crucial components, which convert the applied voltage to motion and generate the required force to move the fluid. The diameter of the piezoelectric layer is smaller than the attached glass. With the application of the electrical potential (*V*) to the piezoelectric wafer with thickness of (*t*), the electric field (ϵ), expressed as =−Vt, makes the wafer shrink in the radial direction with free strain. As a result, a tensile stress and bending moment at the piezo and piezo-glass double layer are exerted, which move the pump membrane to the downward direction. 

The working principle of the piezoelectric micropumps is similar to the reciprocating macro-scale pumps, where both the inlet and outlet are closed during the discharge and suction strokes, respectively. Similar to the macro-scale pumps, where the valves are operating by external forces, there is availability in micro-pumps to utilize those valves, known as active valves [28]. Although mechanically actuated valves are commonly used in macro-scale reciprocating pumps, they are not widespread in micro-pumps because of their complicated fabrication process. Instead, the piezoelectric micro-pumps are able to take the advantage of the passive valves, which can be deformed due to the interaction between the solid and fluid [29]. This feature can adjust the flow rate without using any external force. 

**Figure 1:**
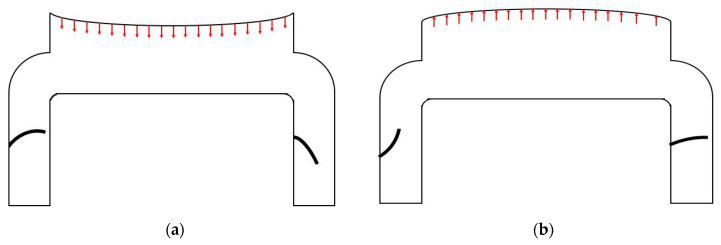
Fully downward and upward motion of the pump membrane. (**a**) Downward motion (**b**) Upward motion.

Figure 1 shows the cross-sectional view of a silicon-based piezoelectric micro-pump, which operates in two-step cycles. The first step starts as the piezoelectric disc moves upwards with respect to the chamber. During this step, the fluid tends to compensate for the vacancy, which is caused by the piezoelectric moving part. Thus, the inlet valves can be easily opened due to the force exerted by the fluid flow. Nevertheless, the outlet valve bends in such a way that it blocks the flow at the outlet, due to the fluid momentum. At the second step, the piezoelectric disc moves downwards with respect to the chamber. As a result, the trapped water inside the channel can leave the channel from the outlet valve. The first and second steps are shown in Figure 1, respectively.

The periodic block voltage is applied on the piezoelectric wafer, which enables us to categorize the pump membrane into two different strokes named as downward and upward. There is an increase in the pressure of the pump chamber when the membrane moves downwards. After switching the voltage off, the pump membrane moves upwards resulting in a decrease in pressure. 

**Figure 2:**
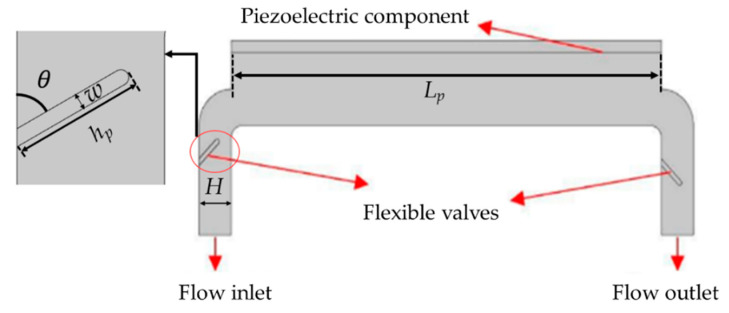
Domain of the piezoelectric pump.

Figure 2 shows the domain of a piezoelectrically driven pump. From a modeling analysis perspective, a piezoelectric pump has three main parts: the working fluid, valves, and piezoelectric material, which is responsible for converting electrical energy into mechanical energy. The mechanical energy at the interface of the piezoelectric wafer results in fluid flow inside the microchannel. Moreover, the interaction between the working fluid and valves results in bending of the valves, whose presence is necessary to avoid symmetry. 

### 2.2. Governing Equations

The governing equations should include fluid flow, elastic behavior of solids, and piezoelectric effect of the material. Thus, they consist of conservation of mass and conservation of momentum. Since the velocity of the fluid is small, the fluid flow is assumed to be laminar. Additionally, due to the small dimensions of the pump, the effect of gravity is neglected. Considering the fact that the working fluid is incompressible, the governing equations for the fluid flow are stated as:

(1) $$\nabla .\left(u-u_{m}\right)=0$$

(2) $$\rho_{f}\partial \left(u-u_{m}\right)/\partial t+\rho_{f}\left(\left(u-u_{m}\right).\nabla \right)\left(u-u_{m}\right)=-\nabla p+\mu_{f}\nabla^{2}\left(u-u_{m}\right)$$

where $\rho_{f}$ is the density of fluid, u is the velocity vector of the fluid, $u_{m}$ is the velocity of the mesh, t is time, p pressure, and $\mu_{f}$ the viscosity of the fluid. The other governing equations consider the bending effect of the valve. As in the fluid domain, due to small size of the valves, the effect of gravity is neglected:

(3) $$\rho_{s}\frac{\partial^{2}d_{s}}{\partial t^{2}}=\nabla .{\left(\Sigma F\right)}^{T}+\rho_{s}f_{b}$$

(4) $$F=I+\nabla .d_{s}$$

where $d_{s}$ is the displacement of solid, F is the applied external force, which is the result of the drag forces, $f_{b}$ is the resulting forces in the body, and $\rho_{s}$ is the density of the solid. Σ is the Piola-Kirchhoff stress tensor, which is related to the Green Lagrangian strain tensor ***G***:

(5) $$\Sigma =2\mu_{s}G+\lambda_{s}tr\left(G\right)I$$

where $\lambda_{s}\;and\;\mu_{s}$ are Lamé constants of the elastic material. They are linked to the Young modulus E and Poisson’s coefficient $\upsilon_{s}$. tr is the tensor trace. $G,\;\lambda_{s}\;and\;\mu_{s}$ are given as:

(6) $$G=\frac{1}{2}\left(F.F^{T}-I\right)$$

(7) $$\lambda_{s}=\frac{\left(\upsilon_{s}E\right)}{\left(1+\upsilon_{s}\right)\left(1-2\upsilon_{s}\right)}$$

(8) $$\mu_{s}=\frac{E}{2\left(1+\upsilon_{s}\right)}$$

The other component of the pump is the piezoelectric material. For pumping the working fluid, an alternating voltage should be applied to the piezoelectric material, which results in strain in the material. The governing equations include the Gauss’s law and elastic behavior of material. The elastic behavior is taken into account in Equations (3)–(8). The charge conservation equations are given as:

(9) ϵ=−∇.V

(10) $$\nabla .\left(D\epsilon \right)=\rho_{v}$$

here, ϵ is the electrical field, V is the applied voltage, $\rho_{v}$ is the charge density, and D is the electrical permittivity of the material. Additionally, assuming the classical beam theory (linear Euler-Bernoulli) with poling the piezoelectric through the thickness, the governing equations of one-dimensional electro-mechanical coupling are as follows:

(11) $$T_{1}=S_{1}e_{11}^{E}-\epsilon_{3}e_{31}$$

(12) $$D_{3}=S_{1}e_{31}-\epsilon_{3}\varepsilon_{33}^{s}$$

In these equations, the subscripts 1 and 3 stand for the directions of the length and thickness of the beam. $T_{1}$ is the stress, and $S_{1}$ is the strain along the length of beam. $D_{3}$ is the electric displacement, and $\epsilon_{3}$ is the electric field through the thickness of the piezoelectric layer. $e_{11}^{E}$ is the elastic stiffness (Young’s modulus) at constant electric field, while $\varepsilon_{33}^{s}$ is the electric permittivity under the constant strain condition, and *e*_31_ is the piezoelectric constant.

The above equations are solved using the FEM (finite element method) and software COMSOL Multiphysics 5.5.

### 2.3. Boundary Conditions and Simulation Algorithm

The boundary conditions are summarized as:

1. First, the voltage is applied to the piezoelectric material, and the displacement and velocity are calculated.
2. The velocity of the piezoelectric material, which is calculated in the previous step, is used as the inlet boundary condition at the interface of the fluid and solid.
3. At the inlet and outlet of the pump, the outlet boundary condition is considered, which results in free movement of water to inside and outside of the channel.
4. The valves are elastic materials, which are assumed to be fixed at the walls.
5. The drag force due to the velocity and pressure field are considered as external forces in the governing equations related to solid mechanics. The drag force is calculated using the software COMSOL Multiphysics 5.5, and the equations are solved using the fully coupled algorithm in COMSOL Multiphysics 5.5.
6. The valves and other boundaries of the channel are considered as wall boundary condition. Moreover, it is worthwhile to state that ALE (Arbitrary Lagrangian and Eulerian) moving mesh is applied to the fluid domain to track the displacement of the valves.

Moreover, the properties of the water are obtained from the COMSOL Library.

### 2.4. Validation of the Model

To avoid computational costs, all the results of the parametric study are provided from 2-D numerical modeling. Even though 2-D simulation results in a 25% underestimation of pressure drop in the pump, with respect to 3-D simulation, it results in reducing the computational time of each solution by more than 40 times.

**Figure 3:**
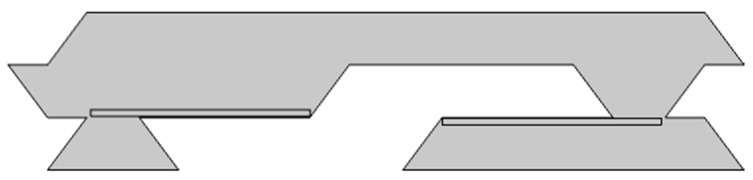
Domain of the piezoelectric pump.

The model presented in the previous section is validated against the experimental data of the study Zengerle et al. [30]. Figure 3 shows the simulated domain. 

Zengerle et al. [30] experimentally studied the effect of the applied frequency to the piezoelectric component. Here, we simulated the corresponding domain under the abovementioned assumptions. The comparison of the numerical results with the experimental data shows that these assumptions result in a maximum error of 14.01% at the applied frequencies between 50 and 400 Hz. 

**Figure 4:**
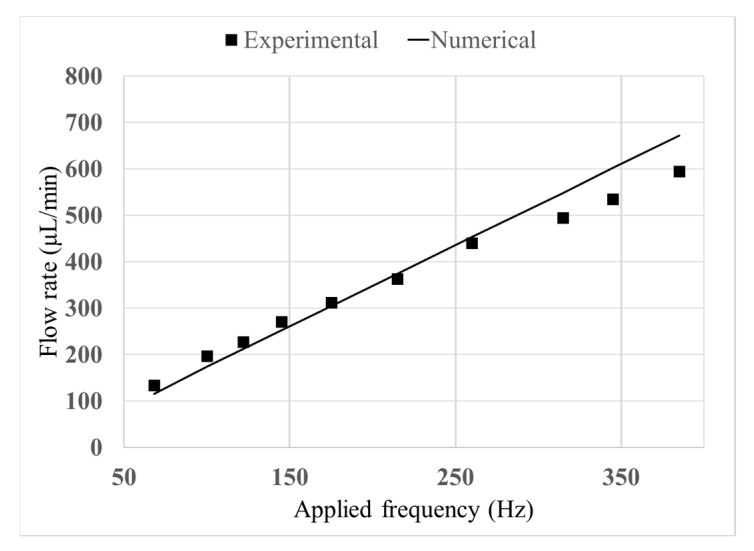
Comparison between experimental [30] and numerical results for the validation of the model.

As can be seen in Figure 4, as the applied frequency increases, the deviation of the model from the experimental data becomes larger. Considering that these assumptions lead to a higher flow rate than the experimental data, this deviation could be due to the increase in the energy loss at the interface, when the applied frequency increases. In this study, the applied frequency to the piezoelectric material is fixed as 50 Hz in all the cases. 

### 2.5. Mesh Convergence

**Figure 5:**
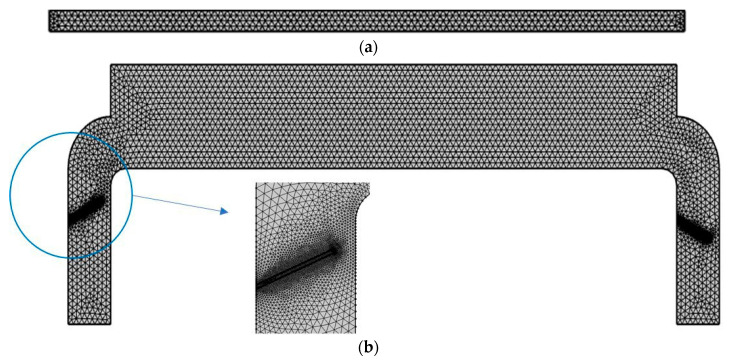

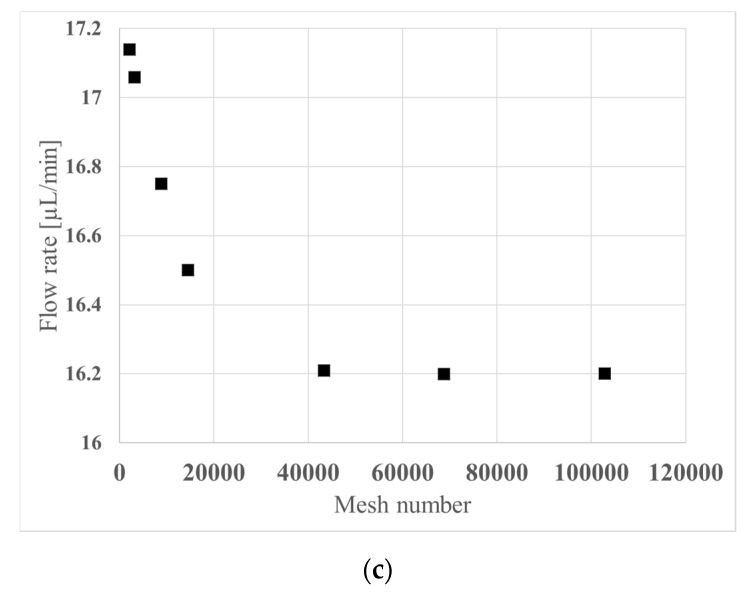
(**a**) Applied meshes to the piezoelectric component (**b**) Applied tetrahedral elements in rest of the simulated models, (**c**) Variation of flow rate for different mesh numbers.

In this study, in order to determine the sensitivity of the grid size to the obtained results, seven different sizes with a total number of cells ranging from approximately 2 × 10^3^ to 10^5^ are used. Figure 5a shows that the simulated model consists of tetrahedral elements for both the fluid and solid domains. In order to perform an accurate simulation, the mesh elements around the valves are finer. The case with 14,530 cells provides a reasonable computation time and results since there is only a 2.4% difference in flow rate between the selected and finest mesh configurations. Figure 5b demonstrates the variation of flow rate with respect to the total mesh numbers. 

## 3. Results and Discussion 

This section presents the behavior of a passive-valve piezoelectric micro-pump during the operation under different conditions. Additionally, the effects of various parameters including the length, width, and attack angle of the valves, piezoelectric length, and applied voltage on the piezoelectric device are displayed. There are inputs, which should be introduced to the model and are summarized in Table 1.

### 3.1. Valve Length and Width

**Figure 6:**
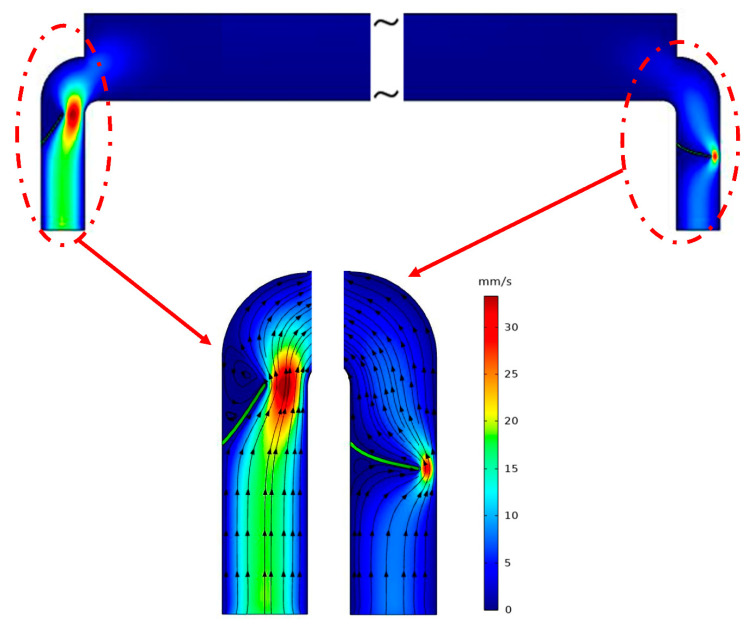
The operation of valve blocking when the piezoelectric component moves upwards (*L*_p_ = 5 mm, *w* = 6 μm, *h*_p_ = 130 μm, *V* = 400 V).

Figure 6 illustrates the velocity field and streamlines of the working fluid for the suction state (i.e., the piezo component at the top). When the piezoelectric component moves upwards, the fluid should be supplied from the left side. Accordingly, the corresponding valve should be under the open condition, which allows the fluid to flow towards the main chamber. However, at the same time, the right-side valve should be closed to confine the reverse flow from the outlet. Moreover, when the piezoelectric component moves downwards, the situation becomes reverse. In other words, in this case, the right-side valve should be open to enable fluid to flow outwards, and the inlet valve should be closed to prevent the fluid flow at the left-side to the inlet. 

**Figure 7:**
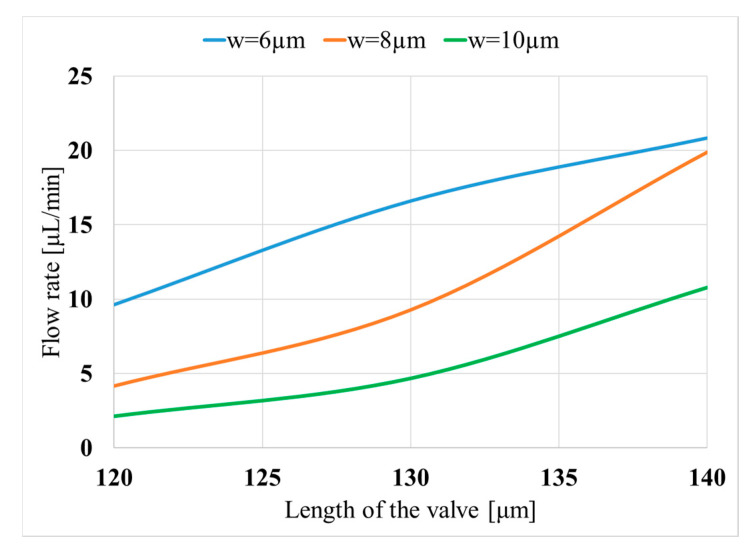
Variation of the obtained flow rate with the valve length (*h*_p_) for three valve widths (w); (*L*_p_ = 5mm, *V* = 400 V).

Figure 7 presents the obtained flow rate for three valve widths (i.e., 6, 8, and 10 µm) and lengths ranging from 120 to 140 µm. As seen in the figure, the efficiency of the piezoelectric pump increases as the length of the valve increases. For instance, for a piezoelectric pump, which has a 5 mm long piezo-component, the achieved flow rates are 2.1, 4.6, 10.7 µL/min for the valve lengths of 120, 130, and 140 µm, respectively, at the applied voltage of 400 V, angle of 45°, and width of 10 µm. 

In order to understand the effect of increasing length on the flow rate, the streamlines shown in Figure 6 should be considered. For the corresponding operation condition, as the length of the valve increases, its performance to block the fluid at the right-side improves. Nevertheless, when the length of the valve increases, the same event occurs at the other side as well. In other words, when the piezoelectric component moves upwards, the longer valve blocks more water at the left-side compared to the shorter valve. Figure 7 demonstrates that the increased blocked region with longer valves is more effective in providing a larger flow rate compared to the configurations with a shorter valve. The mentioned trend is the same for all the cases and is independent of the attack angle and width of the valve, and operation voltage. Therefore, it can be concluded that when the length of the valve increases, the flow rate increases as well.

**Figure 8:**
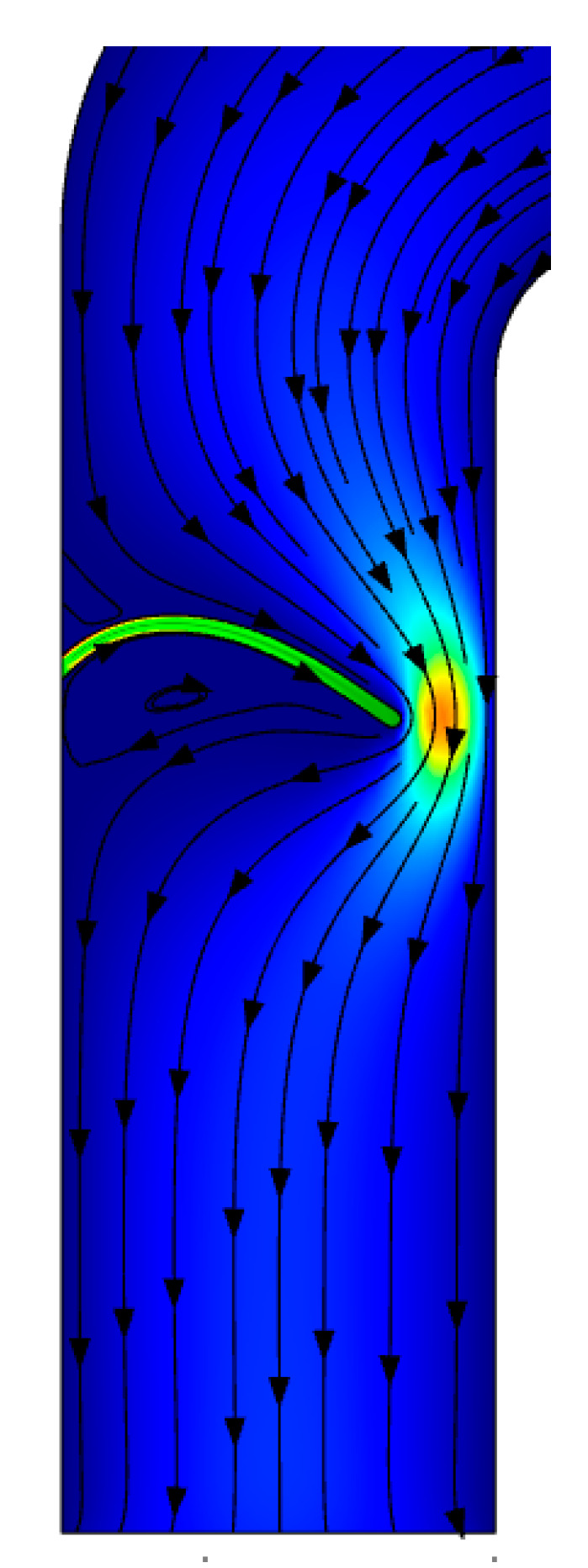
The operation of valve blocking when the piezoelectric component moves downwards (*L*_p_ = 5 mm, *w* = 5 μm, *h*_p_ = 130 μm, *V* = 400 V).

The other parameter is the width of the valve. As the width of the valves increases, its tendency for bending decreases. Therefore, it cannot block the unwanted fluid or efficiently guide the required fluid at the other side of the duct. However, when the width decreases down to a level below a critical value, the valve overbends at the side, it drives the fluid instead of blocking it, as can be seen in Figure 8. 

**Figure 9:**
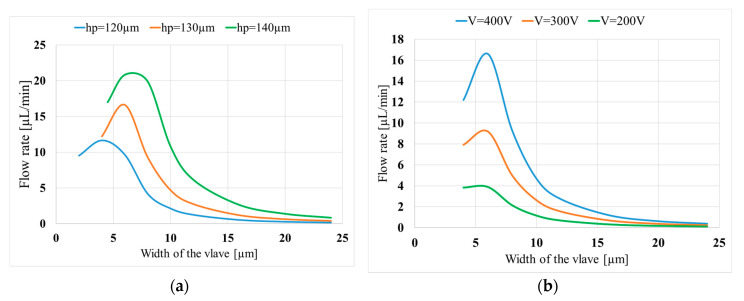
Variation of the obtained flow rate with the valve width (w): (**a**) for three valve lengths (*h*_p_) (*L*_p_ = 5mm, *V* = 400 V); (**b**) for three applied voltages (V) (*L*_p_ = 5mm, *h*_p_ = 130 µm).

Figure 9a shows the effect of the width of the valve on the flow rate for three different valve lengths (i.e., 120, 130, 140 µm). As shown in the figure, there exists a critical value for the width of the valve, where the flow rate is maximum. The optimum width of the valve is 4 µm for the length of 120 µm. The optimum is 6 µm for the lengths of 130 and 140 µm. The optimum width of the valve is dependent on the material as well. As the Young’s modulus of the material decreases, it is expected that the optimum width increases since the valve will move easily.

Figure 9b shows the flow rate with respect to the valve widths for three different voltages (i.e., *V* = 400, 300, and 200 V) and one specific length (*h*_p_ = 130 µm). The optimum valve width corresponds to approximately the same value for all the voltages. Moreover, a very weak relationship can be found between the optimum width and attack angle of the valve. For instance, when the length of the valve is 120 µm and the attack angle changes from 30° to 75°, the obtained optimum width remains as approximately 4 µm for all the cases. Based on the results in Figure 9 and the attack angle dependency, the optimum width of the valve is mainly dependent on the length of the valves rather than other parameters. 

### 3.2. Valve Attack Angle

The attack angle of the valves plays a very crucial role in the efficiency of the pump. As the attack angle increases from 0° to 90°, the applied drag force on the valve increases. However, two drawbacks appear upon the increase in the attack angle. First, the flow will be blocked at the side, where the flow rate is required. Second, the valve might experience overbending as the applied force resulting from the drag forces increases with the attack angle. Our numerical results show that the optimum attack angle can be predicted from the known width and length of the valve.

**Figure 10:**
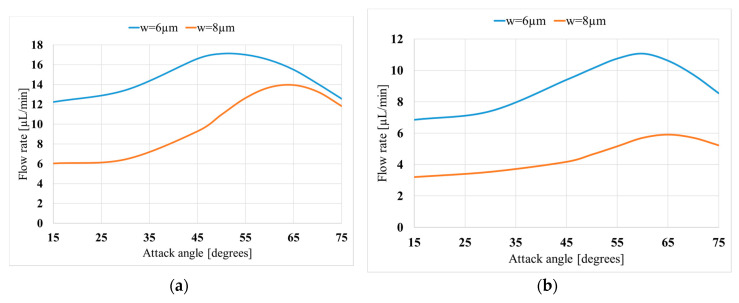
Variation of the obtained flow rate with valve attack angle (θ) for two valve widths (w); (*L*_p_ = 5mm, *V* = 400 V); (**a**) inlet valve; (**b**) outlet valve.

Figure 10 displays the effect of the attack angles of the valves on the flow rate. At the inlet, the optimum attack angle is 55° for the length of 130 µm and the width of 6 µm, which results in a flow rate of 17.1 µL/min. Moreover, when the width is changed to 8 µm, the optimum angle becomes 65°, which results in a flowrate of 14.0 µL/min.

Figure 10b displays the effect of the attack angle of the outlet valve. As can be seen, the changes in the attack angle of the outlet valve can dramatically change the efficiency of the pump. At 200 V of applied voltage, the optimum attack angles are 60° and 65°, for the widths of 6 and 8 µm, respectively. The corresponding flow rates are 11.1 and 5.9 µL/min, respectively. Moreover, for all the cases, the sensitivity analysis shows that the changes in the attack angle do not lead to a dramatic change in the flow rate around the optimum value. For instance, when the attack angle corresponding to the width of 8 µm of the inlet changes from 65° to 60°, the flow rate shrinks from 14.0 to 13.7 µL/m, which corresponds to 1.8% change in the flow rate compared to a 8.3% change in the attack angle.

### 3.3. Piezo Component Length

**Figure 11:**
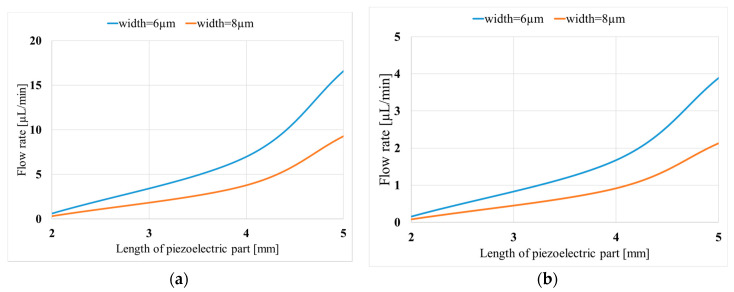
Variation of the obtained flow rate with piezoelectric component length (Lp) for two valve widths (w); (**a**) (*V* = 400 V and *h*_p_ = 130 μm); (**b**) (*V* = 200 V and *h*_p_ = 130 μm).

The other major parameter is the length of the piezo component. Figure 11 presents the supplied flow rate with respect to the length of the piezo component for two valve widths (6 and 8 µm) and applied voltages of 400 and 200 V. Accordingly, as the length of the piezo part increases, the flow rate increases as well because of two reasons. First, as the effective domain of the flow rate increases, the flow rate will increase as well, due to expression $\;Q=\int^{\;}udA$. The other reason is that when the length of the piezoelectric material increases, the displacement increases as well, and the frequency of the AC voltage results in a bigger velocity magnitude at the interface. The change in the velocity at the interface leads to a larger flow rate.

For the applied voltage of 400 V, attack angle of 45°, and width of the valves 6 µm, the flow rates are 0.6, 6.9, and 16.6 µL/min, when the piezoelectric lengths are 2, 4, and 5 mm, respectively.

### 3.4. Applied Voltage

**Figure 12:**
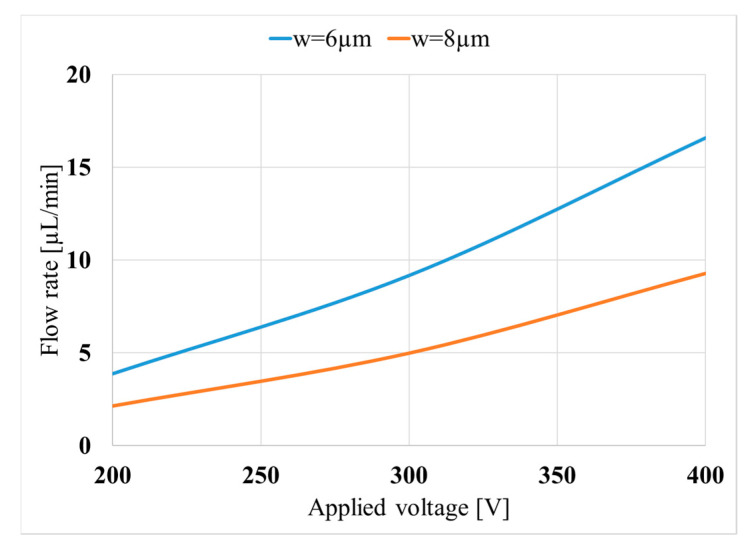
Variation of the obtained flow rate with the applied voltage (*L*_p_) for two valve widths (*w*); (*L*_p_ = 5 mm and *h*_p_ = 130 μm).

Figure 12 shows the effect of the applied voltage. A larger voltage difference results in larger flow rates since bigger displacement at the interface of the fluid and the piezoelectric material can be achieved when applying higher voltages. The results show that the performance of the pump is relatively low at the lower applied voltages. In other words, to achieve considerable flow rates, a relatively high voltage should be applied to the piezoelectric component.

As indicated in the results, the maximum flow rate of the micro-pump depends on the design of its components and operation conditions. The applied 2-D modeling approach enables us to evaluate the effect of these design parameters and operating conditions. Even though the 3-D modeling may provide more accurate results, it does not seem to be very practical when considering the computational time and cost.

### 3.5. Solid Mechanics Analysis

One of the most important features to be considered in the mentioned design is the stress analysis in the valves. Due to the small magnitude of the velocity in the pump and small dimensions of the design (in the order magnitude of micro-meters), the applied stresses on the valves are relatively small. According to the results, as the attack angle of the valves increases, the maximum applied stress grows accordingly. The maximum resulting stress is obtained when the valve length and width are maximum and minimum, respectively. In this case, the corresponding maximum stress is 31.68 kPa for the attack angle of the valve of 75°, applied voltage of 400 V, width and length of the valve of 4 and 140 µm, respectively, and the length of the piezoelectric component of 5 mm.

Based on the obtained low maximum stress, the occurrence of failures due to fatigue after a specific cycle is not expected. Furthermore, in an attempt to propose a potential material to be used as valves, polyester urethanes (PU) could be a suitable candidate [31]. Although the Young’s modulus of this material is around 5 MPa and is approximately 14 times more than the considered value for our computations, considering the necessary strains in the valves, the resulting stress for PU is far away from the failure due to fatigue.

## 4. Conclusions

In this study, the effects of major parameters on the performance of a piezoelectric actuated pump are investigated. The results show that as the length of the valve increases, larger flow rates could be obtained from the piezoelectric micro-pump. Moreover, based on the length, Young’s modulus, and attack angle of the valve, an optimum width can be predicted for the width of the valve. As an important result, the optimum width is almost independent of the flow rate, which ensures us to have optimum flow rates in a wide range of working conditions. Besides, the attack angle of the valve has a critical role in the performance of the pump. The optimum attack angle is dependent on the Young’s modulus, width, and length of the valve. The longer piezoelectric actuator results in bigger displacement and flow area, which results in larger flow rates. Since a larger applied voltage leads to a more energy exchange at the interface, larger flow rates are obtained. Moreover, stress analysis in the valves show that the resulting stresses are relatively small and a failure due to fatigue is not expected.

The implementation of the MEMS technology gives rise to generate high-efficiency microfluidic tools for satisfying the needs in the biomedical industry. The designed piezoelectric micropump in this study can be used in many applications such as local medical treatment and drug delivery systems, where the supply of a controlled flow rate is vital.

## Figures and Tables

**Table 1:** The inputs of the model.

Parameter	Symbol	Value
The height of the channel	*H*	150 µm
Young’s modulus of the valves	*E*	3.6 × 10^5^ (Pa)
The depth of the channel	*D*	400 µm
The length of the valves	*h_p_*	120–140 µm
The width of the valve	*w*	2–24 µm
Applied voltage	*V*	200–400 V
Attack angle	*θ*	15°–75°
Length of piezoelectric component	*L_p_*	2–5 mm
